# Trade-Offs Between Harms and Benefits of Different Breast Cancer Screening Intervals Among Low-Risk Women

**DOI:** 10.1093/jnci/djaa218

**Published:** 2021-01-30

**Authors:** Nicolien T van Ravesteyn, Clyde B Schechter, John M Hampton, Oguzhan Alagoz, Jeroen J van den Broek, Karla Kerlikowske, Jeanne S Mandelblatt, Diana L Miglioretti, Brian L Sprague, Natasha K Stout, Harry J de Koning, Amy Trentham-Dietz, Anna N A Tosteson, the Breast Cancer Surveillance Consortium and the Cancer Intervention and Surveillance Modeling Network

**Affiliations:** 1 Department of Public Health, Erasmus MC University Medical Center, Rotterdam, the Netherlands; 2 Departments of Family and Social Medicine and Epidemiology and Population Health, Albert Einstein College of Medicine, Bronx, NY, USA; 3 Carbone Cancer Center, University of Wisconsin-Madison School of Medicine and Public Health, Madison, WI, USA; 4 Department of Industrial and Systems Engineering, University of Wisconsin-Madison, Madison, WI, USA; 5 Department of Medicine and Department of Epidemiology and Biostatistics, University of California, San Francisco, San Francisco, CA, USA; 6 Department of Oncology, Georgetown University Medical Center and Cancer Prevention and Control Program, Georgetown-Lombardi Comprehensive Cancer Center, WA, USA; 7 Department of Public Health Sciences, UC Davis School of Medicine, Davis, CA, USA; 8 Kaiser Permanente Washington Health Research Institute, Seattle, WA, USA; 9 Department of Surgery and University of Vermont Cancer Center, College of Medicine, University of Vermont, Burlington, VT, USA; 10 Department of Population Medicine, Harvard Medical School and Harvard Pilgrim Health Care Institute, Boston, MA, USA; 11 Department of Population Health Sciences, School of Medicine and Public Health, University of Wisconsin-Madison, Madison, WI, USA; 12 Norris Cotton Cancer Center and the Dartmouth Institute for Health Policy and Clinical Practice, Geisel School of Medicine at Dartmouth, Lebanon, NH, USA

## Abstract

**Background:**

A paucity of research addresses breast cancer screening strategies for women at lower-than-average breast cancer risk. The aim of this study was to examine screening harms and benefits among women aged 50-74 years at lower-than-average breast cancer risk by breast density.

**Methods:**

Three well-established, validated Cancer Intervention and Surveillance Network models were used to estimate the lifetime benefits and harms of different screening scenarios, varying by screening interval (biennial, triennial). Breast cancer deaths averted, life-years and quality-adjusted life-years gained, false-positives, benign biopsies, and overdiagnosis were assessed by relative risk (RR) level (0.6, 0.7, 0.85, 1 [average risk]) and breast density category, for US women born in 1970.

**Results:**

Screening benefits decreased proportionally with decreasing risk and with lower breast density. False-positives, unnecessary biopsies, and the percentage overdiagnosis also varied substantially by breast density category; false-positives and unnecessary biopsies were highest in the heterogeneously dense category. For women with fatty or scattered fibroglandular breast density and a relative risk of no more than 0.85, the additional deaths averted and life-years gained were small with biennial vs triennial screening. For these groups, undergoing 4 additional screens (screening biennially [13 screens] vs triennially [9 screens]) averted no more than 1 additional breast cancer death and gained no more than 16 life-years and no more than 10 quality-adjusted life-years per 1000 women but resulted in up to 232 more false-positives per 1000 women.

**Conclusion:**

Triennial screening from age 50 to 74 years may be a reasonable screening strategy for women with lower-than-average breast cancer risk and fatty or scattered fibroglandular breast density.

There is general consensus that biennial screening from ages 50-74 years is effective in reducing breast cancer mortality and has a favorable balance between benefits and harms (1-3). Risk-based screening has been proposed to improve the efficiency of screening, because it has the potential to lead to a more favorable harm to benefit ratio at the population level (4). Most studies of risk-based screening have focused on women at increased risk (5-7) for whom more intense screening than biennial might be considered. Few studies have assessed the harms and benefits for women at decreased risk (lower than average, ie, a relative risk [RR] < 1). Women with lower-than-average risk of breast cancer are expected to have a less favorable harm to benefit ratio from untargeted screening, suggesting that less intense screening strategies than biennial screening might be appropriate for this group.

The proportion of women at low risk in the population is substantial; for example, 34% of US women aged 40-74 years have a 5-year risk of developing breast cancer below 1.00% based on the Breast Cancer Surveillance Consortium (BCSC) risk model (8,9). Established factors that are associated with substantially decreased risk for breast cancer include fatty breasts, young age at first birth (younger than 20 years), and young age at menopause (younger than 40 years) with relative risks of 0.6-0.7 (10-12); these factors apply to 8%, 12%, and 13% of US women, respectively (10,11). Factors associated with a more modest decrease in risk, such as 3 or 4 full pregnancies (RR = 0.84) and age at menopause between 45 and 49 years (RR = 0.86) (12,13), are even more common, with 39% and 24% of US women aged 50-79 years reporting those factors, respectively (11).

Breast density has also received attention as an important factor that influences risk of developing breast cancer, as well as affecting the balance between benefits and harms of screening, because low breast density not only leads to a reduced risk for developing disease but also increases the sensitivity of mammography (9,10,14). The aim of this study was to assess the benefits and harms of screening by breast cancer risk, breast density, and screening interval among women aged 50-74 years with lower-than-average risk levels using collaborative modeling. Study results are intended to inform discussions about risk-based screening guidelines and practice.

## Methods

### Model Overview

We used 3 well-established microsimulation models developed independently as part of the National Cancer Institute–funded Cancer Intervention and Surveillance Modeling Network consortium: model E (15), model GE (16), and model W (see Table 1) (17). These models have been validated previously (19), have been shown to replicate US population trends in breast cancer incidence and mortality (20,21), and have been used extensively to estimate the impact of different screening scenarios (22‐25). The models and common inputs have been described in detail previously (15‐18,26) (Supplementary Methods, available online).

**Table 1.** Summary of model features^a^

Feature	Model E	Model GE	Model W
Natural history of cancer	Continuous tumor growth	Stage transition	Continuous tumor growth
Details on natural history	Variation in growth rates, includes slow- and fast-growing tumors with varying fatal diameters	All lesions begin as DCIS and can evolve through AJCC-6 stages; variation in dwell times in each stage	Variation in growth rates from nonprogressive disease to hyperaggressive tumors
Tumors obligated to progress	DCIS nonobligate; invasive obligate	DCIS nonobligate; invasive obligate	DCIS and some small invasive are nonobligate; larger invasive obligate
SEER breast cancer data used for model calibration (1975-2010)	Incidence, stage distribution, mortality	Incidence, stage distribution	Incidence and mortality
Screen detection conditioned on	Tumor size, modality, age, density, frequency	Modality, age, density, frequency	Tumor size, modality, age, density, frequency
Implementation of screening benefit	Smaller tumor size	Younger age and earlier stage	Younger age and smaller tumor size
Estimation of overdiagnosis^b^	Difference screen and no screen	Difference screen and no screen	Difference screen and no screen
Implementation of treatment benefit	Cure fraction based on fatal diameter	Hazard reduction	Cure fraction
Factors affecting treatment benefit	ER and HER2; age; year of and size at diagnosis	ER and HER2; age; year of and stage at diagnosis	ER and HER2; age; year of and stage at diagnosis
Model software program^c^	Delphi	C++	C++
Detailed model description	van den Broek et al., 2018 (15)	Schechter et al., 2018 (16)	Alagoz et al., 2018 (17)

a Adapted from (6). Additional information is available from (18), and at https://resources.cisnet.cancer.gov/registry/site-summary/breast/. AJCC = American Joint Committee on Cancer; DCIS = ductal carcinoma in situ; ER = estrogen receptor.

b Overdiagnosis was defined as screen-detected cancer that would not have been diagnosed in a woman’s lifetime in the absence of screening.

c Combined output from all 3 models was analyzed using SAS (Cary, NC) version 9.4.

### Model Inputs

A cohort of US women born in 1970 was simulated using previously described inputs (6,18), such as breast cancer incidence (27), adjuvant therapy (28), and data from the BCSC (http://www.bcsc-research.org) for sensitivity, specificity, and benign biopsy rate of digital mammography by age, breast density, and screening round (first vs subsequent). We modeled 16 subgroups of women, defined on the basis of combinations of risk levels (RR = 0.6, 0.7, 0.85, and 1; see Table 2 for examples of risk factors associated with decreased risk) and 4 breast density categories (Breast Imaging Reporting and Data System categories almost entirely fatty [a], scattered fibroglandular densities [b], heterogeneously dense [c], or extremely dense [d]). The risk level (relative risk) influenced the onset of breast cancer and was assumed to be constant over age. Breast density category was assigned at age 50 years and could decrease by 1 level or remain the same at age 65 years, based on the observed age-specific prevalence in the BCSC. Density affected mammography performance (32), whereas mammography performance was assumed to be unaffected by risk. Risk associated with density (Table 3) was combined multiplicatively with the risk of the different risk levels (relative risks). In this way, density and other risk factors were assumed to be independent determinants of breast cancer risk, consistent with observed data. Thus, a 50-year-old woman with heterogeneously dense breasts and a relative risk of 0.7 had a relative risk of 0.875 (0.7*1.25). In each simulation, women were followed until death or a model-specific upper age of 100 or 120 years. To evaluate the efficacy of different screening scenarios, we assumed 100% uptake of screening and treatment. We modeled biennial screening between ages 50 and 74 years (13 screens) and triennial screening between ages 50 and 74 years (9 screens).

**Table 2.** Factors that are associated with decreased risk for breast cancer reported in literature^a^

Risk estimates	Risk group	Comparison group	Reference
0.65	Age at first birth <20 y	Nulliparity	Ewertz et al., 1990 (13)
0.67	Age at menopause <40 y	Age at menopause 50-54 y	CGHFBC, 2012 (12)
0.69	Age at first birth 20-24 y	Nulliparity	Ewertz et al., 1990 (13)
0.69	5 or more full-term pregnancy	1 or 2 full-term pregnancy	Ewertz et al., 1990 (13)
0.73	Age at menopause 40-44 y	Age at menopause 50-54 y	CGHFBC, 2012 (12)
0.75	Women who breastfed > 12 months	Women who never breastfed	Bernier et al., 2000 (29)
0.78	Women who ever breastfed	Women who never breastfed	Bernier et al., 2000 (29)
0.80-0.81	Age at first birth 25-29 y	Nulliparity	Ewertz et al., 1990 (13); Nelson et al., 2012 (30)
0.82	Age at menarche ≥16 y	Age at menarche = 13 y	CGHFBC, 2012 (12)
0.84	3 or 4 full-term pregnancy	1 or 2 full-term pregnancy	Ewertz et al., 1990 (13)
0.86	Age at menopause 45-49 y	Age at menopause 50-54	CGHFBC, 2012 (12)
0.89	Physical activity for ≥8000 MET min/wk	Physical activity <600 MET min/wk	Wu et al., 2013 (31)
0.87-0.92	Age at menarche at ≥15 y	Age at menarche = 13 y	CGHFBC, 2012 (12); Nelson et al., 2012 (30)

a CGHFBC = Collaborative Group on Hormonal Factors in Breast Cancer; MET = metabolic equivalent.

**Table 3.** Age-specific model input parameters by breast density

Density	Age, y	Density prevalence	Density relative risk^a^
Almost entirely fatty	50-64	0.097	0.5
	≥65	0.135	0.61
Scattered fibroglandular	50-64	0.464	0.84
	≥65	0.533	0.94
Heterogeneously dense	50-64	0.376	1.25
	≥65	0.3	1.28
Extremely dense	50-64	0.063	1.53
	≥65	0.032	1.45

a Age-specific relative risk of breast cancer associated with breast density; reference group is women with average density. Data source: Breast Cancer Surveillance Consortium. The models used sensitivity and specificity by age and screening interval (6).

### Screening Outcomes

For all screening scenarios, we estimated outcomes per 1000 women alive at age 50 years, including the number of ductal carcinoma in situ (DCIS) and invasive breast cancers detected. Benefits included breast cancer deaths averted, life-years gained, and quality-adjusted life-years (QALYs) gained. To calculate QALYs, we applied health-related quality-of-life utilities by age (33), and we applied quality-of-life decrements by attaching weights to specific health states for women undergoing a mammogram and diagnostics (34) and life-years with breast cancer by stage of disease at diagnosis (35). Harms included overdiagnosis, false-positives, and benign biopsies. Overdiagnosis was defined as screen-detected cancer that would not have been diagnosed in a woman’s lifetime in the absence of screening. In addition, harm to benefit ratios (false-positives per life-year gained and overdiagnosis per breast cancer death averted) were calculated.

### Analysis

We presented all outcomes by subgroups of risk and density for each strategy using the median (minimum, maximum) of the 3 models. Each outcome was compared with a reference value, defined as the model-specific results for biennial screening from age 50 to 74 years, all densities combined (thus, with representative population frequencies of breast density categories), and average risk (RR = 1). We evaluated the differences between screening scenarios by assessing the incremental benefits and incremental harms by dividing the incremental harm by the incremental benefits.

We performed sensitivity analyses on varying utility values for undergoing screening and additional workup and on varying specificity by risk (9) (Supplementary Methods, available online).

## Results

### Screening Outcomes

**Figure 1.**
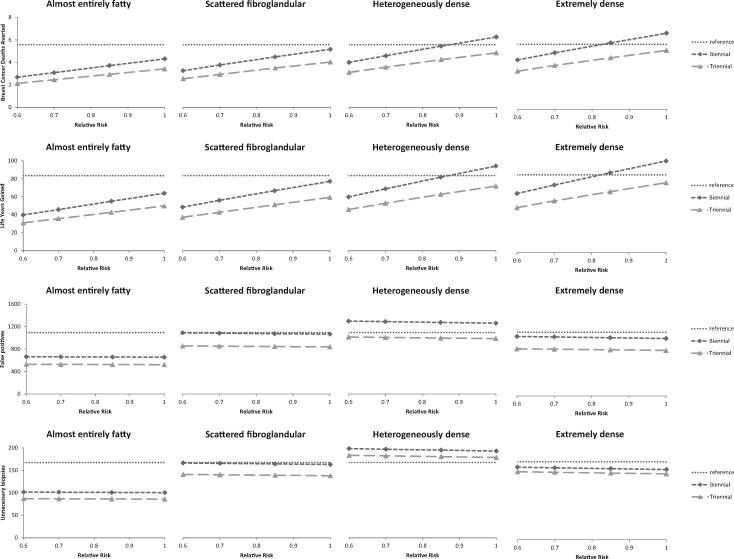
Lifetime benefits and harms from exemplar model (model E). All outcomes are presented per 1000 women followed over their lifetimes by density, relative risk (RR), and screening scenario: breast cancer deaths averted, life-years gained, false-positives, and biopsies. Biennial (diamonds): biennial screening between ages 50 and 74 years (13 screens). Triennial (triangles): triennial screening between ages 50 and 74 years (9 screens). Reference (**dotted horizontal line**) shows the model-specific values for biennial screening from age 50 to 74 years, all densities combined, average risk (RR = 1).

Among 1000 women aged 50 years followed over their lifetimes, the number of invasive breast cancers detected when screening biennially between ages 50 and 74 years varied substantially by subgroup; the highest number of invasive breast cancers was a median of 150 (range across models = 150-177) detected in the average-risk (RR = 1) extremely dense group and decreased with decreasing risk and density in all 3 models to 39 (range = 33-52) in the lowest risk-density category (ie, RR = 0.6 and almost entirely fatty breasts) (Table 4). The trends in lifetime benefits and harms are shown for 1 exemplar model (Figure 1).

**Table 4.** Number of ductal carcinoma in situ (DCIS), invasive breast cancers detected, lifetime benefits, and lifetime harms for biennial screening between ages 50 and 74 years per 1000 women followed over their lifetimes across models^a^

Breast density at age 50 years	Relative risk	No. of DCIS detected, median (min, max)	No. of invasive BCs detected, median (min, max)	Lifetime benefits, median (min, max)			Lifetime harms, median (min, max)		
				No. of BC deaths averted	Life-years gained	QALYs gained	False-positives	Biopsies	Overdiagnosis
Almost entirely fatty	0.60	16 (11, 24)	39 (33, 52)	2.5 (1.6, 2.7)	40 (38, 44)	25 (22, 26)	665 (623, 824)	102 (92, 128)	12 (8, 19)
	0.70	18 (16, 27)	45 (39, 77)	2.9 (1.9, 3.1)	46 (44, 52)	30 (27, 30)	663 (620, 821)	101 (92, 127)	14 (9, 22)
	0.85	22 (20, 32)	54 (47, 101)	3.5 (2.3, 3.7)	55 (53, 62)	38 (33, 38)	659 (617, 816)	101 (91, 126)	17 (11, 26)
	1.00	25 (22, 37)	63 (55, 117)	4.1 (2.7, 4.3)	64 (62, 73)	45 (40, 46)	656 (613, 811)	100 (91, 126)	20 (12, 29)
Scattered fibroglandular density	0.60	22 (12, 27)	60 (58, 64)	3.3 (2.6, 4.3)	60 (48, 76)	36 (29, 47)	1088 (1018, 1267)	166 (151, 197)	17 (11, 18)
	0.70	25 (18, 30)	74 (67, 89)	3.8 (3.0, 4.9)	70 (56, 88)	43 (35, 56)	1083 (1011, 1260)	166 (150, 196)	20 (12, 21)
	0.85	31 (23, 36)	89 (81, 116)	4.5 (3.6, 6.0)	84 (67, 106)	54 (44, 70)	1074 (1001, 1249)	164 (148, 194)	24 (15, 24)
	1.00	36 (25, 41)	103 (94, 133)	5.2 (4.2, 7.0)	98 (77, 124)	65 (52, 84)	1065 (991, 1238)	163 (147, 192)	27 (17, 27)
Heterogeneously dense	0.60	21 (15, 28)	75 (72, 88)	4.0 (3.0, 5.2)	68 (60, 93)	40 (36, 59)	1297 (1213, 1495)	198 (180, 232)	17 (13, 18)
	0.70	24 (21, 32)	102 (87, 106)	4.6 (3.4, 6.0)	79 (69, 108)	49 (44, 71)	1288 (1202, 1484)	197 (178, 230)	19 (15, 20)
	0.85	29 (26, 38)	121 (104, 136)	5.5 (4.2, 7.2)	95 (82, 130)	61 (54, 88)	1274 (1186, 1468)	195 (176, 228)	23 (18, 23)
	1.00	33 (29, 43)	140 (120, 156)	6.3 (4.9, 8.4)	111 (94, 152)	73 (64, 106)	1260 (1170, 1452)	193 (174, 226)	26 (20, 26)
Extremely dense	0.60	18 (17, 30)	94 (83, 95)	4.2 (2.9, 6.3)	65 (63, 113)	41 (40, 76)	1023 (961, 1392)	156 (142, 212)	15 (14, 17)
	0.70	24 (21, 34)	109 (108, 121)	4.9 (3.3, 7.2)	75 (73, 130)	49 (48, 90)	1014 (952, 1379)	155 (141, 210)	17 (15, 19)
	0.85	30 (26, 40)	130 (129, 155)	5.7 (4.0, 8.7)	90 (86, 157)	60 (60, 111)	1001 (938, 1360)	153 (139, 208)	20 (18, 22)
	1.00	33 (30, 45)	150 (150, 177)	6.5 (4.7, 10.1)	106 (98, 182)	72 (70, 131)	988 (924, 1342)	151 (137, 205)	22 (21, 24)

a BC = breast cancer; QALYs = quality-adjusted life-years.

### Benefits

The absolute numbers of lifetime benefits decreased with decreasing risk and with decreasing density in all 3 models. For women with lower-than-average risk and fatty breasts, screening led to fewer benefits (breast cancer deaths averted and life-years gained) than for women at average risk and/or with denser breasts (Table 4; Supplementary Table 1, available online). For example, per 1000 women followed over their lifetime, biennial screening from age 50 to 74 years gained 40 (range = 38-44) life-years in low-risk women (RR = 0.6) with fatty breasts, whereas the same strategy gained a median of 64 (range = 62-73) life-years in average-risk women (RR = 1) with fatty breasts and a median of 106 (range = 98-182) in average-risk women (RR = 1) with extremely dense breast (Table 4). The finding that benefits decreased with decreasing risk (approximately linearly) was consistent across models, screening scenarios, density categories, and outcomes (breast cancer deaths averted, life-years gained, QALYs gained). Absolute benefits also increased with increasing density consistently across models, screening scenarios, and risk groups, although the increase was not linear and showed a leveling off for the highest density category (Table 4; Supplementary Table 1, available online). Biennial screening scenarios resulted in more benefits and triennial screening scenarios in all models and for all risk and density subgroups (Figure 1).

### Harms

**Figure 2.**
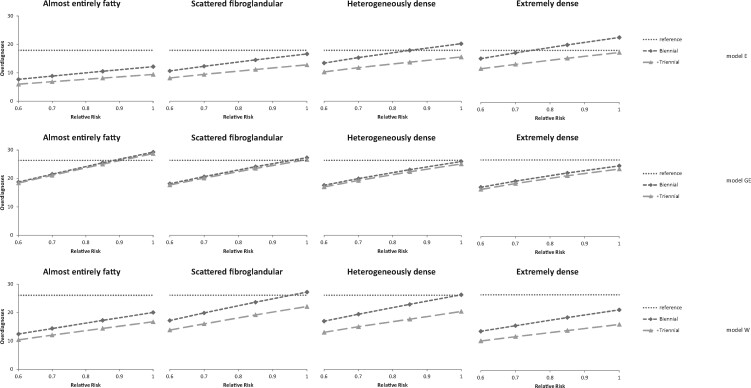
Number of overdiagnosed women per 1000 women aged 40 years followed over their lifetime by density, relative risk (RR), screening scenario, and model: model E (upper part), model GE (middle part), and model W (lower part). Biennial (diamonds): biennial screening between ages 50 and 74 years (13 screens). Triennial (triangles): and triennial screening between ages 50 and 74 years (9 screens). Reference (**dotted horizontal line**) shows the model-specific values for biennial screening from age 50 to 74 years, all densities combined, average risk (RR = 1).

The number of false-positives were relatively stable over risk given our model assumptions (Table 4; Supplementary Table 2, available online), whereas the number of overdiagnoses decreased with decreasing risk (Figure 2). The number of false-positives was highest in breast density category C (heterogeneously dense) (Figure 1). The same trend was found for the number of benign biopsies (Figure 1).

The relationship between overdiagnosis and density varied across models: in model E, overdiagnosis increased with increasing density; in model W, overdiagnosis was highest in the 2 middle categories; and in model GE, overdiagnosis slightly decreased with increasing density (Figure 2). When overdiagnosis was expressed as a percentage of all breast cancers detected, the percentage decreased consistently in all models with increasing density from 22.7% (range = 12.1%-31.9%) to 11.6% (range =10.6%-12.5%) for a relative risk of 1 and did not vary by risk.

### Harm to Benefit Ratios

The ratio between harms and benefits showed diversity across models and measures (Supplementary Table 3, available online). All models predicted a decrease in the number of false-positives per life-year gained with increasing risk and somewhat fewer false-positives per life-year gained in the extremely dense category.

### Screening Scenarios (Biennial vs Triennial)

Biennial vs triennial screening has fewer benefits for the low-risk and low-density subgroups than for average-risk women (Table 5). The additional number of breast cancer deaths averted per 1000 women is 0.4 (range = 0.3-0.6) in women at lowest risk (RR = 0.6) with fatty breasts and 0.6 (range = 0.5-0.7) in women at lowest risk (RR = 0.6) with scattered fibroglandular densities with biennial vs triennial screening. For women with fatty or scattered fibroglandular breast density and a relative risk of 0.6, 0.7, or 0.85, screening biennially (13 screens) vs screening triennially (9 screens) averted less than 1 additional breast cancer death and gained at most 16 life-years and 10 QALYs. For average-risk women with extremely dense breasts, there were 1.5 (range = 1.2-1.5) additional deaths averted, 28 life-years gained, and 19 QALYs gained with biennial vs triennial screening (Table 5).

**Table 5.** The incremental number of breast cancer deaths averted, life-years gained, quality-adjusted life-years (QALYs) gained, false-positives, additional biopsies, and harm to benefit ratios when moving from triennial to biennial screening between ages 50 and 74 years per 1000 women followed over their lifetime

Breast density at age 50 years	Relative risk	Median across models (min, max)								
		No. of additional breast cancer deaths averted	No. of additional life-years gained	QALYs gained	No. of additional false-positives	No. of additional biopsies	No. of additional overdiagnosis	Ratio of additional false-positives per additional life-year gained	Ratio of additional overdiagnosis per additional breast cancer death averted	Ratio of additional screens per additional life-year gained
Almost entirely fatty	0.60	0.4 (0.3, 0.6)	9 (6, 10)	5 (2, 6)	135 (126, 245)	15 (13, 30)	1.7 (0.3, 2.1)	15 (13, 44)	3.1 (1.1, 5.3)	409 (373, 644)
	0.70	0.5 (0.4, 0.6)	10 (6, 11)	6 (3, 7)	135 (125, 244)	15 (12, 30)	2.0 (0.4, 2.3)	13 (11, 38)	3.2 (1.1, 5.2)	356 (318, 558)
	0.85	0.6 (0.5, 0.8)	12 (8, 14)	8 (4, 9)	134 (125, 242)	14 (12, 29)	2.4 (0.5, 2.8)	11 (9, 30)	3.1 (1.0, 5.0)	290 (259, 437)
	1.00	0.6 (0.5, 0.9)	14 (9, 16)	10 (5, 11)	133 (124, 241)	14 (12, 29)	2.7 (0.6, 3.3)	9 (8, 26)	3.1 (1.1, 5.1)	254 (222, 384)
Scattered fibroglandular density	0.60	0.6 (0.5, 0.7)	11 (10, 16)	7 (5, 10)	232 (216, 375)	26 (22, 46)	2.5 (0.4, 3.3)	21 (14, 38)	3.4 (0.8, 5.2)	316 (224, 359)
	0.70	0.7 (0.6, 0.8)	13 (12, 18)	8 (6, 12)	231 (215, 373)	25 (22, 45)	2.9 (0.5, 3.8)	17 (12, 32)	3.4 (0.8, 5.3)	267 (194, 307)
	0.85	0.9 (0.8, 1.0)	16 (14, 21)	10 (8, 14)	229 (212, 369)	25 (22, 45)	3.4 (0.6, 4.5)	15 (10, 27)	3.4 (0.8, 5.1)	226 (163, 254)
	1.00	1.0 (0.9, 1.1)	18 (16, 25)	12 (10, 17)	227 (210, 366)	25 (21, 45)	3.8 (0.7, 5.1)	13 (8, 22)	3.4 (0.8, 4.9)	196 (138, 213)
Heterogeneously dense	0.60	0.8 (0.7, 0.9)	14 (13, 18)	8 (7, 11)	283 (265, 444)	15 (11, 38)	3.1 (0.6, 3.9)	20 (15, 33)	3.5 (0.8, 5.3)	253 (193, 264)
	0.70	0.9 (0.8, 1.0)	16 (16, 21)	10 (9, 14)	281 (262, 441)	15 (11, 38)	3.5 (0.7, 4.4)	18 (12, 28)	3.5 (0.8, 4.9)	219 (165, 226)
	0.85	1.0 (1.0, 1.2)	19 (19, 25)	12 (11, 16)	277 (258, 435)	14 (11, 37)	4.2 (0.8, 5.2)	15 (10, 23)	3.5 (0.7, 5.0)	181 (138, 184)
	1.00	1.2 (1.2, 1.4)	22 (22, 29)	15 (14, 20)	274 (254, 430)	14 (10, 37)	4.7 (0.9, 5.9)	12 (9, 20)	3.3 (0.8, 4.9)	153 (117, 158)
Extremely dense	0.60	1.0 (0.8, 1.0)	18 (15, 18)	10 (10, 12)	213 (200, 423)	10 (7, 36)	3.4 (0.7, 3.5)	14 (11, 24)	3.6 (0.8, 4.5)	199 (198, 231)
	0.70	1.1 (0.9, 1.1)	20 (17, 20)	12 (11, 14)	211 (198, 419)	10 (7, 36)	3.8 (0.8, 4.0)	12 (10, 21)	3.7 (0.8, 4.4)	173 (170, 203)
	0.85	1.3 (1.0, 1.3)	24 (20, 24)	16 (14, 16)	208 (195, 413)	9 (7, 35)	4.5 (1.0, 4.6)	10 (8, 17)	3.6 (0.7, 4.4)	143 (141, 167)
	1.00	1.5 (1.2, 1.5)	28 (23, 28)	19 (16, 20)	205 (192, 408)	9 (7, 35)	5.1 (1.1, 5.2)	9 (7, 14)	3.5 (0.7, 4.2)	120 (120, 145)

The number of additional false-positives was highest for the heterogeneously dense category, lowest for the almost entirely fatty category, and did not vary much by risk. For women with fatty or scattered fibroglandular breast density and a relative risk of no more than 0.85, there were up to 232 additional false-positives per 1000 women (Table 5). There were more additional false-positives per additional life-year gained among the low-risk groups, and this ratio decreased with increasing risk in all models (Table 5). The number of additional overdiagnoses per breast cancer death averted decreased in 2 of the 3 models by risk and density (Table 5). The number of additional screens per additional life-year gained when going from triennial to biennial screening increased with decreasing risk and density consistently across models. In average risk women (RR = 1) with extremely dense breasts, models predicted that 120 (range = 120-145) additional screens were needed to gain 1 life-year when going from triennial to biennial screening, whereas in women at lowest risk (RR = 0.6) with fatty breasts, models predicted a substantially higher number of additional screens needed to gain 1 life-year: 409 (range = 373-644) (Table 5).

### Sensitivity Analysis

Varying utility values for undergoing screening and additional workup or varying specificity by risk did not majorly change the ranking and differences between subgroups (Supplementary Tables 4 and 5, available online).

## Discussion

This is the first collaborative modeling study of breast cancer screening strategies for women at lower-than-average risk, while considering breast density in this assessment. The results indicate that triennial screening from age 50 to 74 years should be considered for women at lower-than-average risk with low density, because this strategy reduces harms while maintaining a large part of the benefits. This conclusion was robust across models and assumptions about disutility associated with screening and variations in specificity by risk.

Our findings are largely in line with previous studies. A previous modeling study, including the same 3 models, focusing on women at increased risk, found that average-risk women with low breast density undergoing triennial screening will maintain a similar or better balance of benefits and harms than average-risk women receiving biennial screening (6). Another modeling study using combined risk-based strategies also found that triennial screening from age 50-74 years was optimal for low-risk and medium-low–risk Spanish women (7) and even investigated less intense strategies (quinquennial screening). Moreover, triennial screening is the currently employed screening frequency in the United Kingdom and has been predicted to lead to a substantial mortality reduction (36). Also, the Canadian Task Force recommends screening with mammography every 2-3 years for women aged 50-69 years (37).

Our results show that for a subgroup of women with a combination of fatty or scattered fibroglandular breast density and low-risk (RR = 0.6, 0.7, 0.85) incremental benefits (deaths averted, life-years gained, and QALYs gained) are small for biennial screening from age 50 to 74 years compared with triennial screening. This is reflected in the higher ratio between additional false-positives and additional life-years gained in the low-risk and low-density subgroups when going from triennial to biennial screening than in the average-risk population, indicating that there are (relatively) more harms relative to benefits in these subgroups than in the average-risk population.

The models consistently found that the benefits of screening decrease with decreasing risk, whereas the number of false-positives and unnecessary biopsies are mostly stable over categories of low risk. The latter was due to our assumption that mammography performance was unaffected by risk. The benefits also decreased with decreasing density, although the decrease in benefits was not so steep when comparing the highest density category to the next category, indicating that elevated risk among women with high density is a more important determinant of absolute screening benefits than high breast density. With regard to harms, false-positives and unnecessary biopsies were highest in the heterogeneously dense category, whereas the trends in overdiagnosis across density categories varied across models.

These results are useful for informing guidelines and for clinical practice. Because the conditions that result in lower-than-average risk are common, primary care providers could use these results in shared decision-making discussions with women. Most risk factors that lead to a decreased risk are not easily modifiable, but they are relatively straightforward to ascertain. If a subgroup of women can be identified to be at low risk, these women can relatively safely decrease their screening intensity from biennial to triennial.

We acknowledge that breast density is not known in women who have never been screened and is therefore difficult to use to tailor the interval of screening among low-risk women. However, it is possible to tailor the screening interval after a first mammogram based on density, especially because mandated standard reporting of breast density to women after a mammogram has become increasingly more common in the United States. Importantly, the measurement of breast density has become more reliable with automated density measures and has similar accuracy in predicting breast cancers (38‐40).

Strengths of this study include consideration of breast density; evaluation of a comprehensive set of outcomes for benefits and harms; and the use of 3 well-established, validated models (19). One of the strengths of collaborative modeling is that the combined results from the different independent modeling groups constitute a sensitivity analysis on model structure. Each model was developed using common data from multiple sources and an elaborate calibration process varying multiple parameters to match population-level breast cancer incidence and mortality data (from Surveillance, Epidemiology, and End Results [SEER]). If models were to include alternative values for standard parameters, they would no longer be calibrated to SEER data, and the resulting predictions could not be viewed as reliable. A strength of our analysis is that each model incorporates different structural assumptions about unobservable natural breast cancer history, including varying assumptions regarding the percent of cancers (invasive and/or DCIS) that do not progress, and sojourn times, which inherently provide a sensitivity analysis on screening benefit. Taken collectively, the cross-model results provide stronger evidence than would any single model varying each parameter individually. In addition, most trends and the ranking of scenarios were very similar across models, except for the overdiagnosis results. We found especially that the trends in overdiagnosis across density categories varied across the models; in model E, the number of overdiagnosed women increases with increasing density, reflecting the higher risk associated with density, whereas in model GE, the number of overdiagnosed women decreased, reflecting the lower sensitivity associated with density, and in model W, overdiagnosis was highest in the 2 middle categories as a result of the 2 opposing causes of higher risk and lower sensitivity. The variation across models reflects uncertainty around overdiagnosis in general and uncertainty around overdiagnosis by density in particular.

Our study also had some limitations. Most importantly, we assumed that the relative risk only influenced the onset of breast cancer and was constant over age. Thus, our models assumed that the age distribution of cancers was similar to the average population reported in SEER and was just proportionately lower. We also assumed that the screening performance and the distribution of tumors in terms of estrogen receptors and HER2 are the same for lower-than-average risk women as that for average-risk women. It would be useful to reassess our results when there are additional data on disease biology and screening performance by risk level. Second, we modeled digital mammography screening. Several studies have suggested that the introduction of tomosynthesis in the United States has led to a reduction in recall rates (41,42), so that the number of false-positives might be reduced if tomosynthesis is widely used. However, the reductions in recall rate are relatively small in the United States (approximately 1%), and the effect of tomosynthesis on other harms, such as overdiagnosis, is still uncertain. In addition, our sensitivity analysis showed that even when quality-of-life effects due to false-positives are not taken into account, the ranking and differences between subgroups were largely unchanged. In addition, our analysis focuses on screening scenarios starting at age 50 years, and results will be different for older starting ages (eg, age 60 years). The absolute risk (for a woman with relative risk of 0.6) is higher at age 60 years than at age 50 years, and therefore more benefits (breast cancer deaths averted) are expected. However, for 60-year-old women, there are fewer life-years to be gained, and overdiagnosis increases by age. Future work might focus on the balance of benefits and harms for starting screening in (low-risk) older women. Finally, the models incorporate different structural assumptions about unobservable natural history, including the following 4 factors. First, the percent of invasive breast cancers that do not progress: model W includes a fraction of tumors with limited malignant potential, whereas models E and GE do not include a subset of invasive cancers that do not regress. Second, the models include a range of nonprogressive DCIS, resulting in a wide range of predicted overdiagnosis of DCIS from 34% to 62% (43). Third, the models assume that the benefit of screening arises from either detection at a smaller tumor size or at an earlier stage, and at a younger age. There is a range between these 3 models in predicted mortality reductions of 25%-32% for biennial screening in ages 50-74 years (22). Finally, for sojourn times, model GE includes an age-dependent sojourn time ranging from 2 to 4 years, whereas models E and W simulate continuous tumor growth with certain distributions, resulting in a wide range of distribution of sojourn times, including a subset of tumors with very short sojourn times as well as very long sojourn times. Estimates of mean sojourn times may be biased if they are based on a model that does not allow for nonprogressive (overdiagnosed) cancer (44).

Despite the substantial differences between models on these key assumptions, models come to the same conclusion regarding the incremental benefits and harms of biennial vs triennial screening in low-risk women.

Overall, our collaborative modeling study showed that triennial screening from ages 50 to 74 years can be considered for women who have fatty or scattered fibroglandular breast density and average or low risk of developing breast cancer and for women with very low risk at any density level. By undergoing more intense screening, these women are subjected to more harms, with only small added benefits. The results contribute to the growing body of evidence that tailored screening has many advantages over age-based guidelines for average populations (7,45). It will be important to translate our findings, and other results, into clinical practice and test the most effective methods for communication of breast cancer risk and breast density to enhance shared decision making about breast cancer screening.

## Funding

This work was supported by the National Cancer Institute at the National Institutes of Health (P01 CA154292, U01 CA199218, U01 CA152958, P30 CA014520, and P30 CA023108). Data collection for model inputs from the Breast Cancer Surveillance Consortium (BCSC) was supported by the National Cancer Institute grant P01 CA154292 and grant U54 CA163303. The collection of BCSC cancer and vital status data used in this study was supported in part by several state public health departments and cancer registries throughout the United States. For a full description of these sources, please see https://www.bcsc-research.org/about/work-acknowledgement.

## Notes

**Role of the funders:** The funders had no role in the design of the study; the collection, analysis, and interpretation of the data; the writing of the manuscript; or the decision to submit the manuscript for publication.

**Disclosures:** Dr Kerlikowske reports unpaid consulting with Grail on the STRIVE study. The other authors have no disclosures.

**Acknowledgment:** The authors thank Isabelle Lefebvre for data processing and analysis.

**Author contributions: **Conception and design: NTvR, CBS, OA, KK, JSM, DLM, BLS, NKS, HJdK, ATD, ANAT; Data analysis and interpretation: NTvR, CBS, JHM, OA, JJvdB, KK, JSM, DLM, BLS, NKS, HJdK, ATD, ANAT; Funding acquisition: KK, JSM, DLM, BLS, ATD, ANAT; Writing-original draft: NTvR; Writing-review&editing: CBS, JHM, OA, JJvdB, KK, JSM, DLM, BLS, NKS, HJdK, ATD, ANAT.

## Data Availability

Input and output data from the models are available by sending a request to the corresponding author of this study (e-mail: n.vanravesteyn@erasmusmc.nl).

## Supplementary Material

djaa218_Supplementary_Data
