# Supplementary material for: Trade-Offs Between Harms and Benefits of Different Breast Cancer Screening Intervals Among Low-Risk Women
Source: J Natl Cancer Inst. 2021 Jan 30;113(8):1017–26. doi: 10.1093/jnci/djaa218 (PMC8502479; doi:10.1093/jnci/djaa218)
Supplement: djaa218_Supplementary_Data [file djaa218_supplementary_data.pdf]

Supplementary Materials

Trade-offs between harms and benefits of different breast cancer screening intervals among low-risk women

Supplementary Methods. Model descriptions.

The models simulate life histories for individual women. Women are born in a breast cancer-free state and some women develop a tumor in a preclinical stage where it can be screen-detected or be diagnosed with breast cancer due to clinical symptoms. Once diagnosed with breast cancer, women receive age-, stage-, and biomarker-specific treatment. On the basis of mammography sensitivity (or thresholds of detection), screening can identify disease in the preclinical period at an earlier stage or smaller size than might occur by clinical detection, possibly resulting in a reduction in breast cancer mortality due to earlier more effective treatment. Women die of either breast cancer or other causes.

Supplementary Methods. Sensitivity analysis.

To account for the variability and uncertainty of the impact of undergoing mammography and having false-positive results on a woman's health-related quality-of-life we performed a sensitivity analysis by varying utility values for undergoing screening and additional work-up. In addition, based on data suggesting that specificity varied somewhat by risk, we evaluated the impact of varying specificity by risk on the number of false-positives.

When short-term negative quality-of-life effects from screening participation and positive test results were not included, the benefits in terms of QALYs gained of screening were somewhat larger in all risk and density groups (Supplementary Table 4). The incremental number of QALYs gained when comparing biennial to triennial screening were slightly higher for the QALYs without adjustment, but the ranking and differences between subgroups remained the same (Supplementary Table 4).

When varying specificity by risk, for women with almost entirely fatty breasts and women with extremely dense breasts, the numbers of false positives decreased slightly for women at lower risk, whereas for women in the middle two density categories, the numbers of false positives increased somewhat (Supplementary Table 5). The differences were, however, small.

Supplementary Table 1. Lifetime benefits (breast cancer deaths averted, life-years gained (LYG), quality-adjusted life years gained (QALYs) gained) for biennial and triennial screening between ages 50-74 years per 1000 women followed over their lifetimes for each model.

Breast Density at age 50 years	Relative Risk	Breast cancer deaths averted		Llife-years gained		QALYs gained	
		Biennial 50-74	Triennial 50-74	Biennial 50-74	Triennial 50-74	Biennial 50-74	Triennial 50-74
Model E[a]							
Almost entirely fatty	0.60	2.7	2.1	40	31	26	20
	0.70	3.1	2.5	46	36	30	24
	0.85	3.7	2.9	55	43	38	30
	1.00	4.3	3.4	64	50	45	36
Scattered fibroglandular density	0.60	3.3	2.5	48	37	29	22
	0.70	3.8	2.9	56	43	35	27
	0.85	4.5	3.5	67	51	44	34
	1.00	5.2	4.0	77	59	52	40
Heterogeneously dense	0.60	4.0	3.1	60	46	36	28
	0.70	4.6	3.6	69	53	44	34
	0.85	5.4	4.2	82	63	54	42
	1.00	6.3	4.8	94	72	64	50
Extremely dense	0.60	4.2	3.3	63	48	41	32
	0.70	4.9	3.8	73	56	49	38
	0.85	5.7	4.4	86	65	60	46
	1.00	6.5	5.1	98	75	70	54
Model GE[b]							
Almost entirely fatty	0.60	2.5	2.2	44	38	25	23
	0.70	2.9	2.6	52	45	30	27
	0.85	3.5	3.1	62	54	38	34
	1.00	4.1	3.6	73	64	46	41
Scattered fibroglandular density	0.60	4.3	3.7	76	66	47	42
	0.70	4.9	4.3	88	76	56	50
	0.85	6.0	5.2	106	92	70	63
	1.00	7.0	6.1	124	107	84	75

Heterogeneously dense	0.60	5.2	4.5	93	80	59	53
	0.70	6.0	5.2	108	93	71	62
	0.85	7.2	6.2	130	111	88	77
	1.00	8.4	7.3	152	130	106	92
Extremely dense	0.60	6.3	5.3	113	95	76	66
	0.70	7.2	6.1	130	110	90	78
	0.85	8.7	7.4	157	132	111	95
	1.00	10.1	8.6	182	154	131	112
Model W[c]							
Almost entirely fatty	0.60	1.6	1.3	38	29	22	16
	0.70	1.9	1.4	44	33	27	19
	0.85	2.3	1.7	53	40	33	24
	1.00	2.7	2.0	62	46	40	29
Scattered fibroglandular density	0.60	2.6	1.9	60	44	36	26
	0.70	3.0	2.2	70	51	43	31
	0.85	3.6	2.7	84	63	54	40
	1.00	4.2	3.2	98	73	65	48
Heterogeneously dense	0.60	3.0	2.2	68	50	40	29
	0.70	3.4	2.5	79	58	49	35
	0.85	4.1	3.1	95	70	61	44
	1.00	4.9	3.7	111	81	73	53
Extremely dense	0.60	2.8	2.1	65	47	40	29
	0.70	3.3	2.4	75	55	48	35
	0.85	4.0	3.0	90	66	60	44
	1.00	4.7	3.5	106	78	72	52

[a]Model-specific reference values: Biennial screening from 50 to 74 for RR=1, when all densities are combined, results in 5.6 breast cancer deaths averted, 84 life-years gained, and 57 QALYs gained.

[b]Model-specific reference values: Biennial screening from 50 to 74 for RR=1, when all densities are combined, results in 8.0 deaths averted, 143 life-years gained, and 99 QALYs gained.

[c]Model-specific reference values*:* Biennial screening from 50 to 74 for RR=1, when all densities are combined, results in 4.4 deaths averted, 101 life-years gained, and 67 QALYs gained.

Supplementary Table 2. Lifetime harms (false positives, biopsies and cases of overdiagnosis) for biennial and triennial screening between ages 50-74 years per 1000 women followed over their lifetimes for each model.

Breast Density at age 50 years	Relative Risk	false positives		biopsies		overdiagnosis	
		Biennial 50-74	Triennial 50-74	Biennial 50-74	Triennial 50-74	Biennial 50-74	Triennial 50-74
Model E[a]							
Almost entirely fatty	0.60	665	530	102	87	7.7	6.0
	0.70	663	528	101	87	8.9	6.9
	0.85	659	525	101	86	10.5	8.2
	1.00	656	522	100	86	12.2	9.4
Scattered fibroglandular density	0.60	1088	856	166	141	10.7	8.2
	0.70	1083	852	166	140	12.3	9.4
	0.85	1074	845	164	139	14.5	11.2
	1.00	1065	839	163	138	16.6	12.8
Heterogeneously dense	0.60	1297	1014	198	184	13.4	10.3
	0.70	1288	1007	197	182	15.3	11.8
	0.85	1274	997	195	180	17.9	13.7
	1.00	1260	986	193	179	20.2	15.6
Extremely dense	0.60	1023	810	156	146	15.1	11.6
	0.70	1014	803	155	145	17.0	13.1
	0.85	1001	793	153	143	19.8	15.2
	1.00	988	783	151	141	22.4	17.2
Model GE[b]							
Almost entirely fatty	0.60	824	579	128	98	18.7	18.4
	0.70	821	577	127	97	21.5	21.1
	0.85	816	574	126	97	25.5	25.0
	1.00	811	571	126	96	29.3	28.7
Scattered fibroglandular density	0.60	1267	892	197	151	18.1	17.7
	0.70	1260	887	196	150	20.6	20.1
	0.85	1249	880	194	149	24.1	23.5
	1.00	1238	872	192	148	27.3	26.6
Heterogeneously dense	0.60	1495	1052	232	194	17.6	17.0

	0.70	1484	1044	230	192	19.9	19.3
	0.85	1468	1033	228	190	23.1	22.3
	1.00	1452	1022	226	189	26.0	25.1
Extremely dense	0.60	1392	968	212	176	16.9	16.2
	0.70	1379	960	210	175	19.0	18.2
	0.85	1360	947	208	172	21.9	20.9
	1.00	1342	935	205	170	24.3	23.2
Model W[c]							
Almost entirely fatty	0.60	623	497	92	80	12.5	10.4
	0.70	620	495	92	79	14.4	12.1
	0.85	617	492	91	79	17.2	14.4
	1.00	613	489	91	79	20.0	16.8
Scattered fibroglandular density	0.60	1018	801	151	129	17.2	13.8
	0.70	1011	796	150	128	19.9	16.0
	0.85	1001	789	148	127	23.6	19.2
	1.00	991	781	147	126	27.2	22.1
Heterogeneously dense	0.60	1213	948	180	169	17.0	13.0
	0.70	1202	940	178	167	19.4	15.0
	0.85	1186	928	176	165	22.9	17.7
	1.00	1170	916	174	163	26.3	20.4
Extremely dense	0.60	961	761	142	135	13.5	10.2
	0.70	952	753	141	134	15.4	11.7
	0.85	938	743	139	132	18.3	13.8
	1.00	924	732	137	130	20.9	15.9

[a]Model-specific reference values: Biennial screening from 50 to 74 for RR=1, when all densities are combined, results in 1094 false positives, 167 unnecessary biopsies and 17.9 overdiagnoses.
[b]Model-specific reference values: Biennial screening from 50 to 74 for RR=1, when all densities are combined, results in 1335 false positives, 207 unnecessary biopsies and 26.3 overdiagnoses.
[c]Model-specific reference values*:* Biennial screening from 50 to 74 for RR=1, when all densities are combined, results in 1018 false positives, 151 unnecessary biopsies and 26.1 overdiagnoses.

Supplementary Table 3. Harm-benefit ratios (false positives per life-year gained and overdiagnosis per breast-cancer death averted) for biennial and triennial screening between ages 50-74 years per 1000 women for each model.

**Breast Density at age 50 years**	**Relative Risk**	**false positives per life-year gained**		**overdiagnosis per breast-cancer death averted**	
		**Biennial 50-74**	**Triennial 50-74**	**Biennial 50-74**	**Triennial 50-74**
Model E[a]					
Almost entirely fatty	0.60	16.7	17.1	2.9	2.8
	0.70	14.5	14.8	2.9	2.8
	0.85	12.0	12.3	2.8	2.8
	1.00	10.3	10.5	2.8	2.7
Scattered fibroglandular density	0.60	22.5	23.1	3.3	3.2
	0.70	19.3	20.0	3.3	3.2
	0.85	16.1	16.5	3.2	3.2
	1.00	13.8	14.2	3.2	3.2
Heterogeneously dense	0.60	21.7	22.1	3.4	3.3
	0.70	18.8	19.1	3.3	3.3
	0.85	15.6	15.9	3.3	3.2
	1.00	13.4	13.7	3.2	3.2
Extremely dense	0.60	16.1	16.8	3.6	3.5
	0.70	14.0	14.5	3.5	3.5
	0.85	11.7	12.1	3.5	3.4
	1.00	10.0	10.4	3.4	3.4
Model GE[b]					
Almost entirely fatty	0.60	18.7	15.1	7.5	8.4
	0.70	15.9	12.8	7.4	8.2
	0.85	13.1	10.6	7.2	8.1
	1.00	11.2	9.0	7.1	8.0
Scattered fibroglandular density	0.60	16.7	13.6	4.3	4.8
	0.70	14.4	11.7	4.2	4.7
	0.85	11.8	9.6	4.1	4.5

	1.00	10.0	8.1	3.9	4.4
Heterogeneously dense	0.60	16.0	13.1	3.4	3.8
	0.70	13.7	11.3	3.3	3.7
	0.85	11.3	9.3	3.2	3.6
	1.00	9.6	7.9	3.1	3.5
Extremely dense	0.60	12.3	10.2	2.7	3.1
	0.70	10.6	8.7	2.6	3.0
	0.85	8.7	7.2	2.5	2.8
	1.00	7.4	6.1	2.4	2.7
Model W[c]					
Almost entirely fatty	0.60	16.2	17.3	7.6	8.3
	0.70	14.0	15.1	7.6	8.4
	0.85	11.6	12.4	7.5	8.4
	1.00	9.9	10.6	7.5	8.3
Scattered fibroglandular density	0.60	16.9	18.1	6.7	7.2
	0.70	14.5	15.5	6.7	7.2
	0.85	11.9	12.6	6.6	7.0
	1.00	10.1	10.7	6.4	6.9
Heterogeneously dense	0.60	17.9	19.1	5.7	5.9
	0.70	15.3	16.3	5.6	5.9
	0.85	12.5	13.3	5.5	5.7
	1.00	10.6	11.3	5.4	5.6
Extremely dense	0.60	14.8	16.1	4.7	4.8
	0.70	12.6	13.7	4.7	4.8
	0.85	10.4	11.2	4.6	4.6
	1.00	8.7	9.4	4.5	4.6

[a]Model-specific reference values: Biennial screening from 50 to 74 for RR=1, when all densities are combined, results in 13.1 false positives per life-year gained and 3.2 overdiagnoses per breast-cancer death averted.
[b]Model-specific reference values: Biennial screening from 50 to 74 for RR=1, when all densities are combined, results in 9.4 false positives per life-year gained and 3.3 overdiagnoses per breast-cancer death averted.
[c]Model-specific reference values*:* Biennial screening from 50 to 74 for RR=1, when all densities are combined, results in 10.1 false positives per life-year gained and 5.9 overdiagnoses per breast-cancer death averted.

Supplementary Table 4. The incremental number of QALYs gained with and without* adjustments for screening and work-up when moving from triennial to biennial screening between ages 50-74 years per 1000 women followed over their lifetime for each model.

Breast Density at age 50	Relative Risk	QALYs gained with adjustments			QALYs gained without adjustments*		
		Model E	Model GE	Model W	Model E	Model GE	Model W
Almost entirely fatty	0.60	5.5	2.2	6.0	6.9	4.5	7.4
	0.70	6.5	2.9	7.3	7.9	5.2	8.7
	0.85	8.2	4.2	9.2	9.7	6.6	10.6
	1.00	9.5	5.1	10.8	11.0	7.5	12.3
Scattered fibroglandular density	0.60	6.6	4.6	10.0	8.9	8.0	12.1
	0.70	8.2	5.9	11.7	10.5	9.3	13.9
	0.85	10.0	7.8	14.2	12.2	11.2	16.4
	1.00	11.8	9.8	17.1	14.0	13.2	19.2
Heterogeneously dense	0.60	8.2	6.8	11.3	10.9	10.8	13.9
	0.70	9.8	8.6	13.6	12.6	12.6	16.2
	0.85	12.3	11.3	16.5	15.0	15.2	19.0
	1.00	14.9	13.6	19.8	17.6	17.5	22.3
Extremely dense	0.60	9.8	10.4	11.5	11.9	14.2	13.6
	0.70	11.3	12.4	13.6	13.4	16.2	15.7
	0.85	13.9	15.8	16.5	16.0	19.6	18.5
	1.00	16.2	19.0	19.8	18.3	22.7	21.8

* the number of QALYs without adjustments do not include disutility of screening and work-up (the impact of undergoing mammography and having false-positive results on a woman's health-related quality-of-life)

Supplementary Table 5. The number of false positives assuming specificity to be independent on risk or to vary by risk, when moving from triennial to biennial screening between ages 50-74 years per 1000 women followed over their lifetimes for each model.

**Breast Density at age 50**	**Relative Risk**	**False positives (specificity stable over risk)**			**False positives (specificity varies by risk)**		
		**Model E**	**Model GE**	**Model W**	**Model E**	**Model GE**	**Model W**
Almost entirely fatty	0.60	135	245	126	129	233	120
	0.70	135	244	125	129	232	120
	0.85	134	242	125	128	232	119
	1.00	133	241	124	133	241	124
Scattered fibroglandular density	0.60	232	375	216	249	402	232
	0.70	231	373	215	232	374	216
	0.85	229	369	212	226	365	209
	1.00	227	366	210	227	366	210
Heterogeneously dense	0.60	283	444	265	300	471	281
	0.70	281	441	262	291	457	271
	0.85	277	435	258	275	431	255
	1.00	274	430	254	274	430	254
Extremely dense	0.60	213	423	200	208	414	196
	0.70	211	419	198	209	416	197
	0.85	208	413	195	214	427	201
	1.00	205	408	192	205	408	192
